# Lack of the Sodium-Driven Chloride Bicarbonate Exchanger NCBE Impairs Visual Function in the Mouse Retina

**DOI:** 10.1371/journal.pone.0046155

**Published:** 2012-10-09

**Authors:** Gerrit Hilgen, Antje K. Huebner, Naoyuki Tanimoto, Vithiyanjali Sothilingam, Christina Seide, Marina Garcia Garrido, Karl-Friedrich Schmidt, Mathias W. Seeliger, Siegrid Löwel, Reto Weiler, Christian A. Hübner, Karin Dedek

**Affiliations:** 1 Department of Neurobiology, University Oldenburg, Oldenburg, Germany; 2 Institute of Human Genetics, University Hospital Jena, Jena, Germany; 3 Division of Ocular Neurodegeneration, Centre for Ophthalmology, Institute for Ophthalmic Research, University of Tübingen, Tübingen, Germany; 4 Institut für Allgemeine Zoologie und Tierphysiologie, Friedrich-Schiller-Universität Jena, Jena, Germany; University Zürich, Switzerland

## Abstract

Regulation of ion and pH homeostasis is essential for normal neuronal function. The sodium-driven chloride bicarbonate exchanger NCBE (*Slc4a10*), a member of the SLC4 family of bicarbonate transporters, uses the transmembrane gradient of sodium to drive cellular net uptake of bicarbonate and to extrude chloride, thereby modulating both intracellular pH (pH_i_) and chloride concentration ([Cl^−^]_i_) in neurons. Here we show that NCBE is strongly expressed in the retina. As GABA_A_ receptors conduct both chloride and bicarbonate, we hypothesized that NCBE may be relevant for GABAergic transmission in the retina. Importantly, we found a differential expression of NCBE in bipolar cells: whereas NCBE was expressed on ON and OFF bipolar cell axon terminals, it only localized to dendrites of OFF bipolar cells. On these compartments, NCBE colocalized with the main neuronal chloride extruder KCC2, which renders GABA hyperpolarizing. NCBE was also expressed in starburst amacrine cells, but was absent from neurons known to depolarize in response to GABA, like horizontal cells. Mice lacking NCBE showed decreased visual acuity and contrast sensitivity in behavioral experiments and smaller b-wave amplitudes and longer latencies in electroretinograms. Ganglion cells from NCBE-deficient mice also showed altered temporal response properties. In summary, our data suggest that NCBE may serve to maintain intracellular chloride and bicarbonate concentration in retinal neurons. Consequently, lack of NCBE in the retina may result in changes in pH_i_ regulation and chloride-dependent inhibition, leading to altered signal transmission and impaired visual function.

## Introduction

The vertebrate retina represents a neuronal tissue with a high metabolic rate. Thus, regulation of intracellular pH (pH_i_) is of vital importance because energy metabolism is a proton-producing process [1]. Although light-evoked retinal activity leads to changes in pH large enough to influence retinal circuits, e.g. by modulating gap junctional networks [2] or voltage- and ligand-gated ion channels [3], pH regulation in the retina is only partially characterized. The sodium bicarbonate co-transporter NBCn1 contributes to pH regulation in photoreceptors and lack thereof causes blindness [4] as does a lack of the chloride bicarbonate anion exchanger AE3 which was shown to be expressed in Müller cells and horizontal cells [5], [6]. However, the role of NCBE in the retina has never been studied. The sodium-driven chloride bicarbonate exchanger NCBE (*Slc4a10*) uses the sodium transmembrane gradient to intrude bicarbonate and extrude chloride [7], [8]; though some studies suggest a role as an electroneutral sodium bicarbonate co-transporter (NBCn2) [9]. There exist different splice variants of NCBE/NBCn2 with different sites of expression [8], [10], [11]. In the mouse, NCBE is broadly expressed in the brain [7], [12], [13] and disruption of *Slc4a10* leads to impaired pH_i_ regulation in hippocampal neurons and increased seizure thresholds [12].

As a potential chloride extruder, NCBE may also contribute to the regulation of the intracellular chloride concentration ([Cl^−^]_i_) [7], [8] in retinal neurons. The sodium-potassium-chloride co-transporter NKCC1 was shown to maintain a high [Cl^−^]_i_ in retinal ON bipolar cell dendrites and horizontal cells so that GABA induces a depolarization in these compartments [14]. In contrast, the electroneutral potassium chloride co-transporter KCC2 represents the major active chloride extruder in neurons. It was shown to be expressed in OFF bipolar cells and in ON bipolar cell axon terminals, keeping [Cl^−^]_i_ low so that GABA or glycine induce an inhibitory current [14], [15]. Thus, in bipolar cell dendrites, differential expression of KCC2 and NKCC1 chloride transporters serves to generate GABA-evoked responses of different polarity [16]. As NCBE expression precedes KCC2 expression in other brain areas [13], NCBE may also contribute to neuronal chloride extrusion in the retina.

Using immunostaining and confocal microscopy we show that NCBE is strongly expressed in the retina, namely in bipolar cells and amacrine cells. With markers for individual bipolar and amacrine cell types, we reveal a differential expression in ON and OFF bipolar cell compartments and starburst amacrine cells in which NCBE is colocalized with KCC2. To analyze the functional importance of NCBE, we used a NCBE-deficient mouse line [12] and show that lack of NCBE leads to altered retinal responses and impaired visual performance. Whether these effects are caused by an altered pH_i_ regulation of retinal neurons, by changes in the driving force for inhibitory currents or by a combination of both, remains to be seen.

## Results

### Expression of NCBE in the Mouse Retina

**Figure 1:**
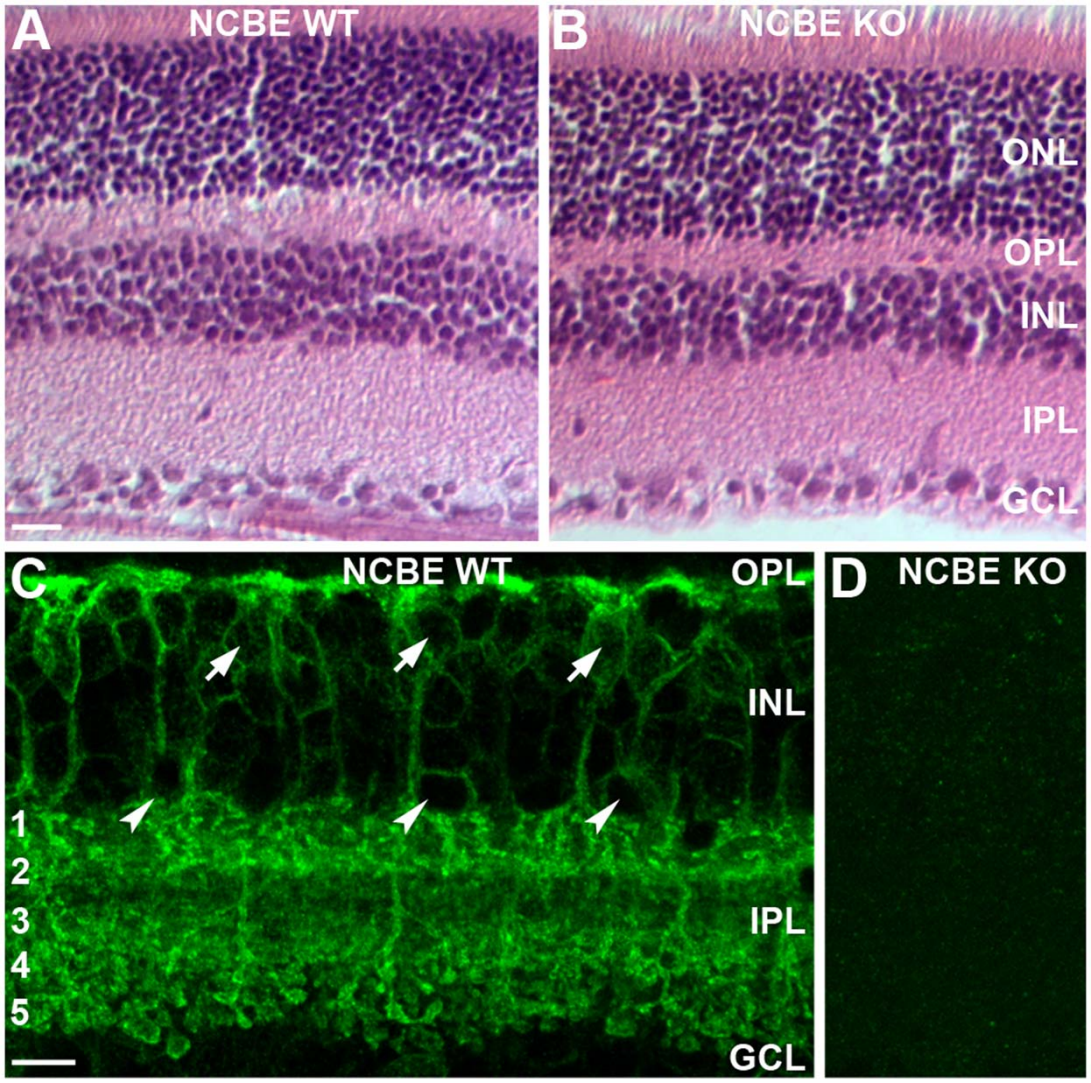
NCBE is strongly expressed in the mouse retina. *A, B,* H/E stainings of NCBE WT (A) and KO (B) retinal sections. Gross morphology of the retina did not differ between both genotypes. ***C, D,*** Projections (5 µm) of NCBE WT (***C***) and KO (***D***) retinal sections stained for NCBE (green). Bipolar (arrows) and amacrine (arrowheads) cells express NCBE, with a strong expression in both plexiform layers (***C***). No NCBE expression was found in the retina of NCBE KO mice (***D***), confirming the specificity of the antibody. Numbers 1–5 are labeling the IPL strata, scale bars = 10 µm.

The sodium-driven chloride bicarbonate exchanger NCBE is broadly expressed in the brain, including choroid plexus, cortex, olfactory bulb, cerebellum, brainstem, spinal cord, and the retina [12], [13]. We first examined if the retina of NCBE-deficient mice shows gross morphological changes. However, H/E stainings of NCBE WT (Fig. 1A) and NCBE KO retina (Fig. 1B) revealed no obvious morphological changes in the NCBE KO retina. Somata sizes of retinal neurons and widths of the plexiform layers appeared normal (Fig. 1B).

To further characterize NCBE expression in the retina, we stained retina sections of NCBE KO and WT mice with a polyclonal antibody against NCBE [12]. NCBE was predominantly present in both plexiform layers of the retina (Fig. 1C). Photoreceptors and their terminals were devoid of label (Fig. S1) whereas some cell membranes in the distal and proximal inner nuclear layer (INL) were stained for NCBE, presumably representing bipolar (Fig. 1C, arrows) and amacrine cell somata (Fig. 1C, arrowheads). No NCBE staining was found in NCBE KO retina sections, indicating that the NCBE antibody was specific for the mouse retina (Fig. 1D). However, as the antibody used in our study is expected to detect all different splice variants, it remains unclear which variants are expressed in the retina.

### NCBE is Differentially Expressed in Bipolar Cell Compartments

Double stainings for NCBE and the vesicular glutamate transporter 1 (VGluT1), a marker for bipolar cell terminals [17], showed that all axon terminals of ON and OFF bipolar cells in the IPL were stained for NCBE (not shown). As murine bipolar cells comprise five different OFF and seven different ON bipolar cell types [18], we double-labeled NCBE WT retina sections with NCBE and specific bipolar cell markers to analyze the NCBE expression in individual subtypes.

**Figure 2:**
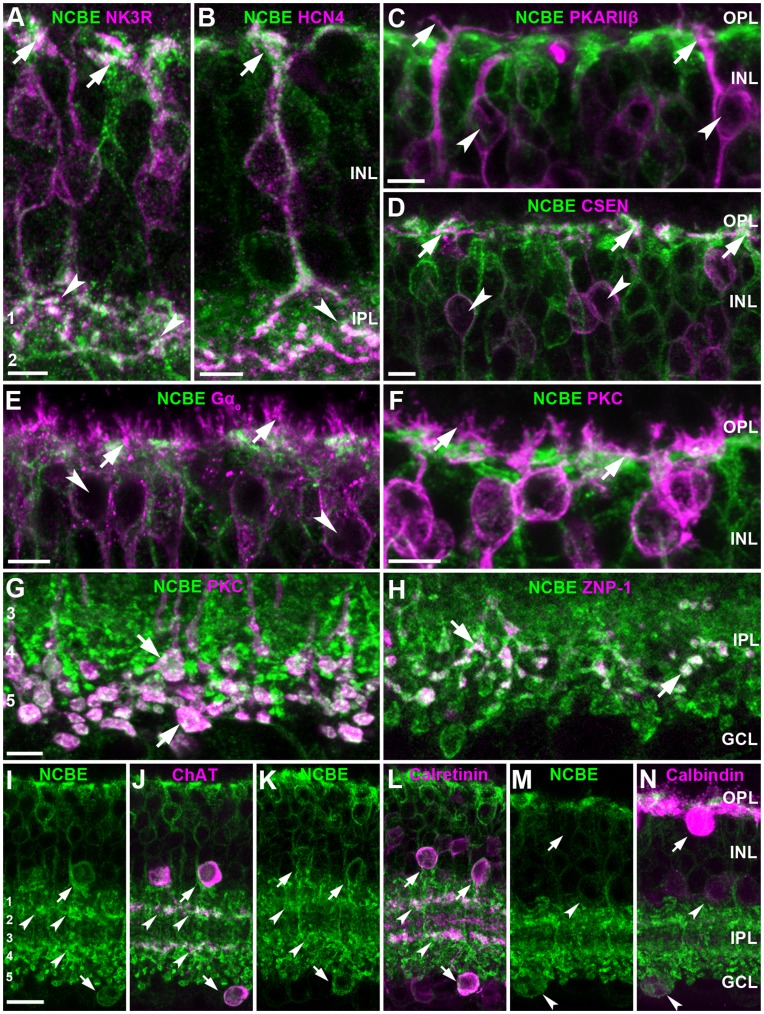
NCBE is expressed in bipolar cells and amacrine cells. ***A,*** NCBE (green) is expressed in axon terminals (arrowheads) and somata (arrows) of type 1 and/or 2 OFF bipolar cells, labeled with NK3R (magenta). ***B,*** HCN4-labeled type 3A OFF bipolar cells (magenta) showed NCBE (green) distributed on dendrites (arrows) and axon terminals (arrowheads). ***C, D,*** PKARIIβ-labeled type 3B OFF bipolar cells (***C***, magenta) and CSEN-labeled type 4 OFF bipolar cells (***D***, magenta) with NCBE (green). Only proximal (arrows) but not distal dendrites (arrowheads) of type 3B OFF bipolar cells showed NCBE expression (***C***). Dendrites (arrows) and somata (arrowheads) of CSEN-labeled type 4 OFF bipolar cells were also positive for NCBE (***D***). ***E,*** Gα0-labeled dendrites (magenta, arrows) of ON bipolar cells did not show NCBE expression (green). However, some ON bipolar cell somata (arrowheads) were NCBE-positive. ***F, G,*** PKCα-labeled rod bipolar cells dendrites (magenta) showed no NCBE expression (**F**, green, arrows), whereas axon terminals strongly express NCBE (***G***, arrows). ***H,*** ZNP-1-labeled axon terminals (magenta) of type 6 ON bipolar cells showed NCBE expression (green, arrows). ***I, J,*** Retinal sections of NCBE WT mice were stained for NCBE (***I, J***, green) and ChAT (***J*** *,* magenta). Merging the NCBE channel (***I***) with the ChAT channel (***J***) revealed that somata (arrows) and dendrites (arrowheads) of ChAT-labeled starburst amacrine cells express NCBE. ***K, L,*** NCBE- (***K, L*** *,* green) and calretinin-labeled retinal sections (***L*** *,* magenta). NCBE expression was found on calretinin-labeled dendrites (arrowheads) and somata (arrows) of amacrine cells in the retina. ***M, N,*** Calbindin-labeled horizontal cells (arrows) do not express NCBE, but calbindin-labeled somata (arrowheads) of amacrine cells do. All images represent projections (3 µm) of confocal stacks. Numbers 1–5 are labeling the IPL strata, scale bars (*A–H*) = 5 µm; (*I–M*) = 10 µm.

Type 1 and 2 OFF bipolar cells can be labeled by antibodies against NK3R [18]. Double staining with NCBE showed strong NCBE expression on dendrites and axon terminals of NK3R-positive OFF bipolar cells (Fig. 2A). OFF type 2 cells can also be stained by antibodies against synaptotagmin II (ZNP-1) [18], [19] and double labeling revealed that somata and axon terminals of ZNP-1-positive type 2 cells intensely expressed NCBE (not shown).

OFF bipolar cell types 3A and 3B can be distinguished based on their immunoreactivity to antibodies against the ion channel HCN4 and the protein kinase A regulatory subunit IIβ (PKARIIβ), respectively [18], [20]. Double stainings for HCN4 and NCBE showed that NCBE was expressed predominantly in dendrites (Fig. 2B, arrow) and axon terminals (Fig. 2B, arrowhead) of OFF bipolar cell type 3A. However, NCBE was not expressed in PKARIIβ-labeled dendrites (Fig. 2C, arrows) and somata (Fig. 2C, arrowheads) of OFF bipolar cell type 3B, confirming existing evidence that type 3A and 3B cells differ in membrane protein composition [20].

Type 4 OFF bipolar cells are immunoreactive to calsenilin (CSEN), a calcium-binding protein [18]. Double-labeling revealed that NCBE was expressed in dendrites (Fig. 2D, arrows) but not in somata (Fig. 2D, arrowheads) of type 4 OFF bipolar cells. As antibodies against CSEN and PKARIIβ also label amacrine cells, NCBE expression on axon terminals of OFF bipolar cell types 3B and 4 could not be determined with these markers alone. However, VGluT1- and NCBE-labeled retina sections showed full colocalization in layer 2 of the IPL (not shown), in which the terminals of type 3B and 4 OFF bipolar cells stratify, confirming that all OFF bipolar cells expressed NCBE in their terminals.

To analyze NCBE expression on ON bipolar cells, we double-stained the retina with antibodies against NCBE and the G-protein subunit Gα_o_, which is a marker for rod and cone ON bipolar cells [21]. However, dendrites (Fig. 2E, arrows) and somata (Fig. 2E, arrowheads) of ON bipolar cells were devoid of label. This was confirmed by double stainings for NCBE and PKCα, a marker for rod bipolar cells [18]. Again, no NCBE immunoreactivity was found on dendrites of rod bipolar cells (Fig. 2F, arrows).

In contrast, PKCα-labeled axon terminals of rod bipolar cells were intensely labeled with NCBE (Fig. 2G, arrows). This was confirmed using antibodies against CaB5, a marker for type 3, 5 and rod bipolar cells [22]. CaB5/NCBE double staining also revealed NCBE on axon terminals of type 5 ON bipolar cells (data not shown). Additionally, NCBE localized to type 6 ON bipolar cell axon terminals (Fig. 2H, arrows) as shown by double staining with antibodies against ZNP-1 [18], [19].

Taken together, we show that a deletion of NCBE did not alter the morphology of the retina and that NCBE was expressed in OFF bipolar cell dendrites and in ON and OFF bipolar cell axon terminals.

### NCBE is Expressed in Starburst Amacrine Cells

Beside the expression of NCBE in bipolar cells, we also found NCBE-labeled cells in the proximal INL (Fig. 1C), presumably on amacrine cells. To examine NCBE expression in these interneurons, we tested colocalization of NCBE with specific amacrine cell markers.

Choline acetyltransferase (ChAT) is predominantly expressed by starburst amacrine cells in the mouse retina. These cells are mirror-symmetrically organized with somata in the INL and the ganglion cell layer (GCL) and are involved in direction selectivity of ganglion cells [23]. Double labeling revealed that somata of ChAT-labeled starburst cells in the INL and GCL express NCBE (Fig. 2I, J, arrows). Also the dendrites in the cholinergic layers 2 and 4 of the IPL were occasionally labeled for NCBE (Fig. 2I, J, arrowheads).

In the mouse retina, antibodies against calretinin label amacrine and ganglion cells [21]. NCBE immunoreactivity was found on calretinin-labeled dendrites in the cholinergic layers 2 and 4 of the IPL (Fig. 2K, L, arrowheads) and on calretinin-labeled somata in the proximal INL (Fig. 2K, L, arrows). These cells may again represent starburst cells as these cells are also calretinin-positive [21]. We found no NCBE immunoreactivity on calretinin-labeled ganglion cell somata or on dendrites in layer 3 of the IPL.

To test for NCBE expression in horizontal cells, we used antibodies directed against calbindin, which also label amacrine and ganglion cells [21]. Double staining revealed that horizontal cells (Fig. 2M, N, arrow) and ganglion cell somata do not express NCBE, whereas calbindin-labeled amacrine and displaced amacrine cells do (Fig. 2M, N, arrowheads).

### NCBE is Coexpressed with KCC2

**Figure 3:**
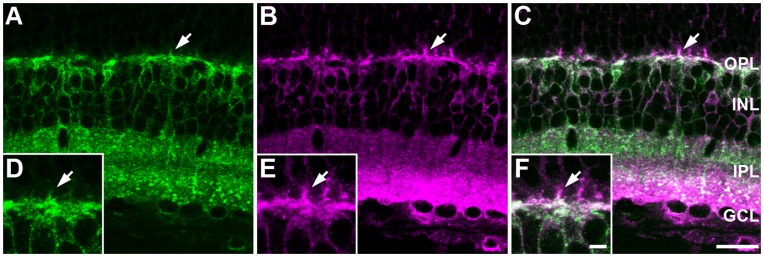
NCBE is colocalized with KCC2. *A–C,* Projections (3 µm) of NCBE WT retinal sections stained for NCBE (***A, C*** *,* green) and the chloride extruder KCC2 (***B***, ***C***, magenta). Higher magnification of single scans (0.5 µm) of NCBE WT retinal sections double-stained for NCBE (***D, F***, green) and KCC2 (***E,*** ** ***F*** *,* magenta) revealed that KCC2 and NCBE are expressed on the same cellular compartments in the retina (arrows). Scale bar = 10 µm.

Because NCBE may function as a pH_i_ regulator and chloride extruder [7], [12] in the retina, we compared the immunoreactivity pattern of NCBE with immunoreactivity pattern of KCC2, another neuronal chloride extruder [15]. KCC2 is involved in the generation of an axo-dendritic chloride gradient in retinal ON cone bipolar cells [14], [16] and plays a role in the direction selectivity of starburst amacrine cells [24], [25]. Maximum (Fig. 3A–C) and single scan projections (Fig. 3D, E) of double stainings for KCC2 and NCBE showed that both transporters are fully colocalized on the same bipolar cell compartments: OFF bipolar cell dendrites and ON and OFF bipolar cell axon terminals.

**Figure 4:**
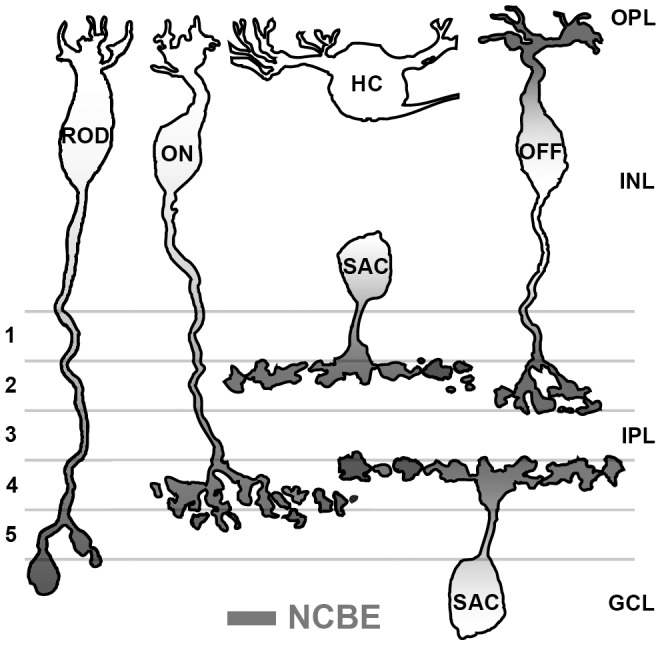
Summary of the NCBE expression in the mouse retina. NCBE is expressed (grey) on the axon terminals of rod ON, cone ON and OFF bipolar cells and on the dendrites of OFF bipolar cells. NCBE is not expressed in dendrites of ON bipolar cells and OFF bipolar cell type 3B. NCBE is also expressed in starburst amacrine cells (SAC), but not in dendrites of ganglion cells (GC). NCBE is also not expressed in photoreceptors (not shown) and horizontal cells (HC).

The expression of NCBE in the mouse retina is summarized in Figure 4. Our results revealed that NCBE was differentially expressed in bipolar cell compartments and is also expressed in starburst amacrine cells. NCBE was not expressed in horizontal cells (Fig. 4) and photoreceptors (not shown). Whether NCBE was expressed in ganglion cell dendrites could not be determined unequivocally. Interestingly, NCBE was expressed in the same retinal cell compartments as KCC2. Thus, NCBE may play a similar role in maintaining a low [Cl^−^]_i_ and contribute to direction selectivity in the retina. As NCBE may also regulate pH_i_ in retinal neurons, these findings prompted us to analyze the physiological consequences of NCBE deletion.

### Electroretinography in NCBE-deficient Mice Indicates ON Bipolar Cell Dysfunction

**Figure 5:**
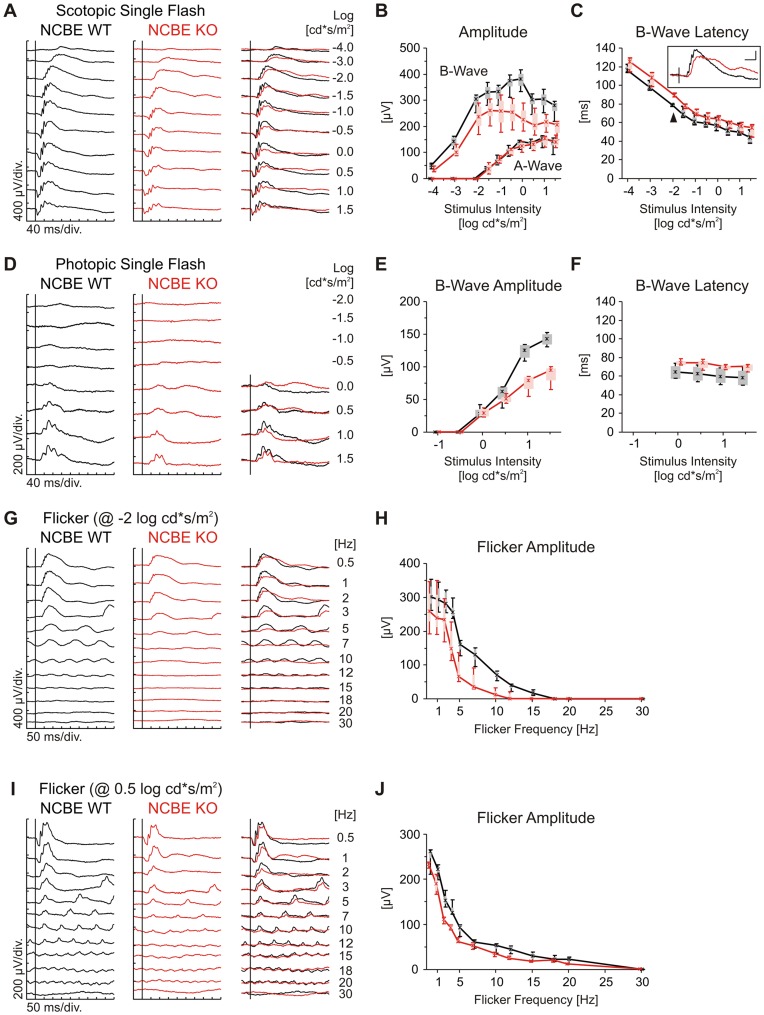
Electroretinography in NCBE-deficient mice indicates ON bipolar cell dysfunction. *A, D,* Representative single flash ERG recordings from NCBE WT (black) and KO (red) mice (age 12 months) for increasing intensities under dark-adapted (***A***, scotopic) and light-adapted (***D***, photopic) conditions. ***B, C, E, F,*** Box-and-whisker plots of single flash ERG b-wave amplitudes (***B, E***) and latencies (***C, F***), plotted against flash intensity. Scotopic b-wave but not a-wave amplitudes in NCBE KO mice were reduced (***A,*** ** ***B***), and b-wave latencies (***C***) were increased compared to NCBE WT mice. *Inset* in ***C***: Overlay of scotopic single flash ERG response traces of NCBE WT (black) and NCBE KO (red) mice at −2.0 log cd*s/m^2^ intensity (arrow head). Scale bar: horizontal 50 ms, vertical 100 µV. Under photopic conditions, b-wave amplitudes (***D, E***) and b-wave latencies (***F***) of NCBE KO mice were similarly affected. ***G,*** ** ***I,*** Representative ERG recordings of a flicker frequency series (flash intensity ***G***: −2 log cd*s/m^2^; ***I***: 0.5 log cd*s/m^2^) under scotopic conditions. Flicker amplitudes (***H, J***) of NCBE KO mice decreased at much lower flash frequencies than that of WT controls. Vertical lines an ***A***, ***D***, ***G***, ***I*** represent stimulus onset. In all quantitative plots (***B, C, E, F, H, J***), boxes indicate the 25% and 75% quantile range, whiskers indicate the 5% and 95% quantiles, and solid lines connect the medians of the data. NCBE WT (n = 2), NCBE KO (n = 3).

The functional status of the retina in health and disease may be examined via electroretinography (ERG). Although the ERG is a mass response, several protocols varying stimulus intensity and frequency as well as the light environment (background) allow to obtain detailed insights in the functionality of rod and cone photoreceptors and their downstream neurons as long as transient and not spiking signals are generated [26], [27]. Here, we compared ERGs of NCBE KO and WT mice at ages of 12 months (PM12) to avoid potential confounding by the differences in birth weight mentioned below. Figure 5A shows the scotopic single flash ERG responses from dark-adapted NCBE KO (red) and WT (black) mice stimulated with increasing light intensities (−4.0 to 1.5 log cd*s/m^2^). We observed no difference in the scotopic single flash ERG a-wave, an initial negative deflection after light stimulation, between NCBE KO and WT mice (quantification Fig. 5B, lower part). Since the a-wave, when reaching saturation before the onset of the b-wave, reflects rod photoreceptor function in mice, this finding indicates that lack of NCBE had no measurable effect on the maximal output of rod photoreceptors, which is in agreement with a lack of NCBE expression in photoreceptors of WT mice.

Scotopic b-wave amplitudes of NCBE KO mice were smaller than those in NCBE WT mice at rod-specific intensities (below −2.0 log cd*s/m^2^) and at higher stimulus intensities, at which both rod and cone photoreceptors are activated (Fig. 5A, B, upper part). Also, the b-wave latencies of NCBE KO mice were increased for all intensities under scotopic conditions and the waveforms were prolonged (Fig. 5C, inset). Together with the unchanged a-wave (Fig. 5B, lower part), this indicates either an impaired synaptic transmission to bipolar cells or a problem in the ON bipolar cells themselves, for instance a reduced excitability.

The slight amplitude reduction plus waveform prolongation was also observed in the scotopic flicker ERGs (0.5 to 30 Hz) at a constant intensity of −2 log cd*s/m^2^ (Fig. 5G). In particular, the waveform prolongation led to a reduced flicker fusion frequency, i.e. NCBE KO flicker amplitudes were only mildly decreased at low stimulus frequencies (0.5 to 3 Hz) compared to WT, but rapidly declined at higher frequencies (5–10 Hz), and were practically zero at frequencies above 10 Hz (Fig. 5G, H).

The cone system responses were assessed with the photopic single flash ERG (Fig. 5D). Similar to scotopic conditions, NCBE KO b-wave amplitudes were considerably decreased at high stimulus intensities (1 and 1.5 log cd*s/m^2^) and b-wave latencies were increased compared to WT mice (Fig. 5E, F).

Finally, we also obtained some information about cone ON and OFF systems via scotopic (dark-adapted) flicker ERGs (0.5 to 30 Hz) at a constant intensity of 0.5 log cd*s/m^2^ (Fig. 5I, J). In this paradigm, the responses are dominated by the rod system below about 3–5 Hz, by the cone ON system from about 5–15 Hz, and by the cone OFF system at higher frequencies. We found small, but consistent amplitude reductions in the frequency ranges attributed to the rod and cone ON system, respectively, but not in the range attributed to the cone OFF system (Fig. 5I, J). This is surprising given the strong NCBE expression in OFF bipolar cells.

In addition to ERG recordings of PM12 mice, we also recorded ERGs of NCBE KO and WT mice at the age of 4 weeks (PW4). The results (Fig. S2) were similar to that of NCBE KO PM12 mice shown here. Furthermore, we morphologically evaluated the PW4 mice *in vivo,* immediately after ERG recordings, and confirmed that the gross organization of the retina was not altered due to a genetic deletion of NCBE (Fig. S3). Yet, the weight of NCBE KO mice was significantly reduced compared to the WT at PW4 (not shown). Thus, we decided not to use this data here, as the observed differences in ERG amplitudes and latencies may have resulted from a developmental delay in NCBE KO mice in this group. However, as we could reproduce the results in the PM12 mouse group and such adult NCBE KO have a normal weight, lifespan [12] and eye size, impairments in PM12 ERGs most likely originate from altered retinal signal transmission rather than developmental defects.

### Ganglion Cell Responses of NCBE-deficient Mice Show Temporal Changes

The intense expression of NCBE on bipolar and amacrine cells together with the impaired ERGs indicated that a deletion of NCBE may alter retinal signal processing, especially in the ON pathway. To test this, we recorded extracellular ganglion cell light responses and compared them between genotypes.

**Figure 6:**
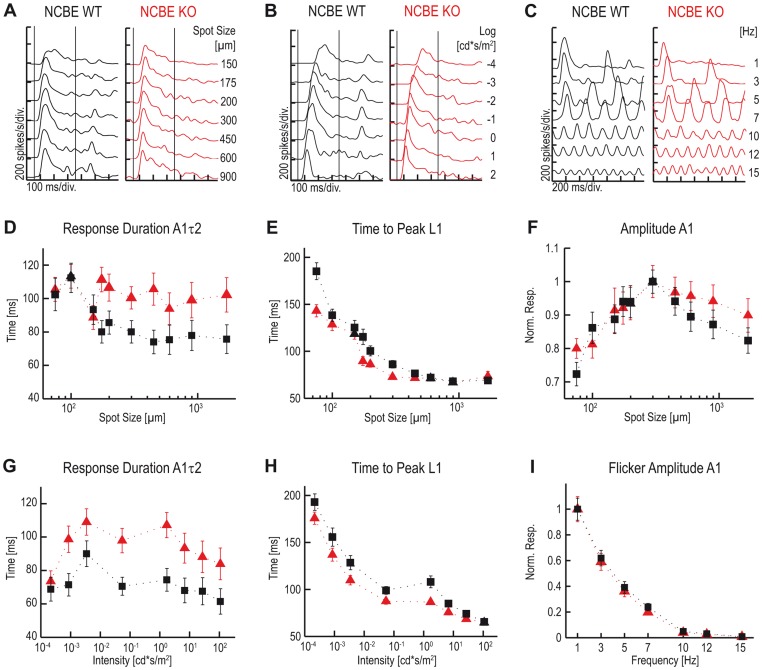
Light responses of ON-transient ganglion cells are longer and faster in NCBE-deficient mice. ***A–C,*** Representative single PSTH light responses of representative NCBE KO (red) and WT (black) ON-transient ganglion cells responding to stimuli with increasing spot sizes (***A***), increasing light intensities (***B***) and increasing frequencies (***C***). ***D, G,*** The response durations *(A1τ2)* of NCBE KO ON-transient ganglion cells compared to NCBE WT ON-transient ganglion cells were increased when either the spot size (***D***; RM two-way ANOVA: F (1, 59) = 6.68; p = 0.0123) or the intensity (***G***; RM two-way ANOVA: F (1, 59) = 7.05; p = 0.0102) was increased. ***E, H,*** The time to peak *(L1)* of NCBE KO ON-transient ganglion cells was decreased compared to NCBE WT ON-transient ganglion cells for both stimuli (***E***; RM two-way ANOVA: F (1, 59) = 7.67; p = 0.0075; ***H***; RM two-way ANOVA: F (1, 59) = 4.83; p = 0.0319). ***F,*** NCBE KO and WT ON-transient ganglion cell responses *(A1)* of a spot size series were normalized and KO response amplitudes were larger for stimuli >300 (***F***; RM two-way ANOVA: F (1, 59) = 2.81; p = 0.1449). ***I,*** Normalized cell responses *(A1)* of a flicker series showed no significant differences (***I***; RM two-way ANOVA: F (1, 59) = 0.02; p = 0.8866). Vertical lines an ***A*** and ***B*** represent stimulus onset and offset, respectively. Values are presented as mean ± standard error of the mean (SEM). NCBE WT (n = 31), NCBE KO (n = 30).

First, we compared the light responses of ON-transient ganglion cells. For all ganglion cells measured, different response parameters were extracted from peri-stimulus time histograms (PSTHs; see Methods; Fig. 6): response amplitude *(A1)*, response duration *(A1τ2)*, and time to peak *(L1)*. Figure 6 shows single PSTHs of representative ON-transient ganglion cells from NCBE KO (red) and WT mice (black) responding to stimuli with a constant light intensity (6.9 cd*s/m^2^) and increasing spot size (75–1,700 µm; Fig. 6A), a constant spot size (300 µm) and increasing light intensity (−4 to 2 log cd*s/m^2^; Fig. 6B), and a constant spot size (300 µm) and light intensity (6.9 cd*s/m^2^) with increasing temporal frequencies (1–15 Hz; Fig. 6C), respectively.

At a constant light intensity (6.9 cd*s/m^2^), response duration of NCBE KO ON-transient ganglion cells was increased for spot sizes above 175 µm (*A1τ2*; Fig. 6D, n = 30) and differed significantly from the NCBE WT (n = 31; for ANOVA values, please refer to the legend of Fig. 6), pointing to more sustained responses in KO cells. The time to peak of NCBE KO ON-transient ganglion cells was significantly decreased for stimuli with increasing spot sizes (*L1*; Fig. 6E). Also, in the NCBE KO, response amplitudes were larger for stimuli >300 µm (*A1*, Fig. 6F). Thus, spatial tuning is altered in NCBE KO mice. However, the average spot size eliciting the maximum firing rate was 300 µm for both genotypes and was in line with the suggested receptive field center size of ganglion cells [28], [29].

This spot size (300 µm) was used for stimulations with increasing light intensity. Under these conditions, response amplitudes were not significantly different (not shown) but NCBE KO ON-transient ganglion cells responded significantly longer (*A1τ2*; Fig. 6G) and faster (*L1*, Fig. 6H). As the impaired ERG b-wave rather suggested a slowing of responses, we stimulated ganglion cells with a flicker series (1–15 Hz; Fig. 6C). The PSTH parameter *A1* was taken as a measure for the cells’ response. However, response amplitudes did not differ between NCBE WT and KO mice (Fig. 6I). These data suggest that ON-transient ganglion cells respond stronger, longer and faster in NCBE-deficient mice despite the profound changes found in ON bipolar cell responses by ERGs.

**Figure 7:**
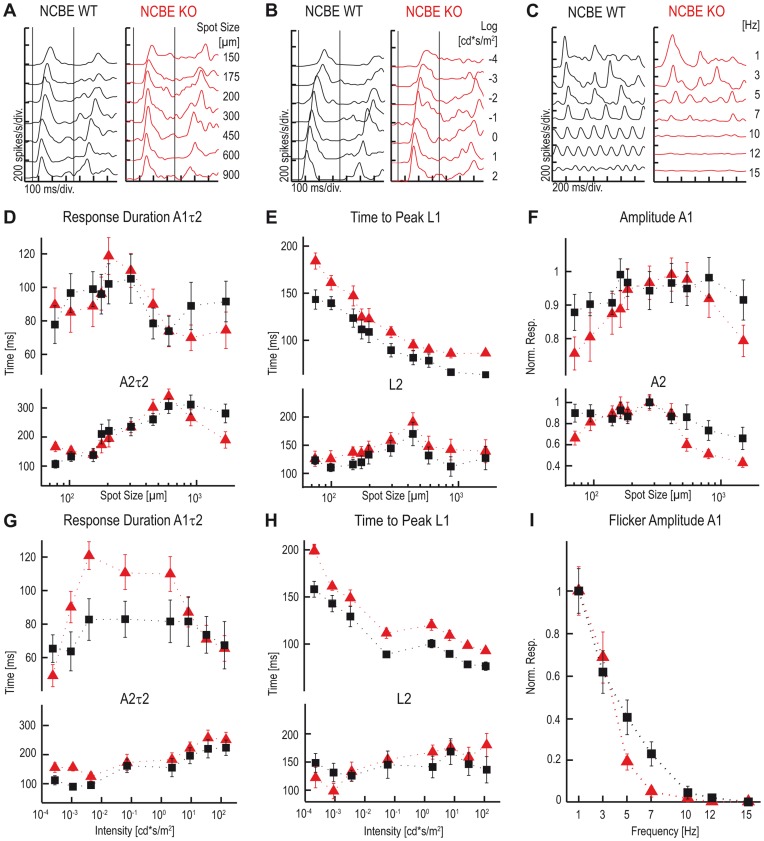
Light responses of ON-OFF ganglion cells are slower in NCBE-deficient mice. **A–C,** Single PSTH of representative NCBE KO (red) and WT (black) ON-OFF ganglion cell light responses, evoked with increasing spot sizes (***A***), light intensities (***B***) and stimulus frequencies (***C***). ***D, G,*** No significant differences were found between the ON *(A1τ2)* and OFF(*A2τ2)* response durations of WT and KO cells when spot size was increased [***D***; top (ON): F(1, 29) = 0.06; p = 0.8141; bottom (OFF): F(1, 29) = 0.01; p = 0.9425]. However, when light intensity was increased, response durations were longer in NCBE KO mice [***G***; top (ON): F(1, 29) = 5.98; p = 0.0207; bottom (OFF): F(1, 29) = 4.50; p = 0.0426]. ***E, H*** **,** Time to peak of the ON response *(L1*) but not of the OFF response (*L2*) was significantly increased in NCBE KO when either the spot size [***E***; Top (ON): F(1, 29) = 6.13; p = 0.0194; bottom (OFF): F(1, 29) = 1.94; p = 0.1741] or the intensity were increased [***H***; top (ON): F(1, 29) = 12.56; p = 0.0014; bottom (OFF): F(1, 29) = 0.53; p = 0.4736]. ***F,*** No significant differences were found between the normalized ON (*A1*) and OFF response amplitudes (*A2*) with increasing spot size (***F***; top (ON): F(1, 29) = 0.03; p = 0.8565; bottom (OFF): F(1, 29) = 0.28; p = 0.5978). ***I,*** Normalized cell responses *(A1)* of a flicker series. NCBE KO cells showed decreased responses and were not able to follow higher frequencies comparably to NCBE WT cells (***I***; F(1, 29) = 2.94; p = 0.0969). Vertical lines an ***A*** and ***B*** represent stimulus onset and offset, respectively. Values are presented as mean ± SEM. Statistical values were obtained from repeated measurement ANOVA. NCBE WT (n = 16), NCBE KO (n = 15).

Next, we compared the light responses of ON-OFF ganglion cells in both genotypes. Single PSTHs of representative cells are shown in Figure 7A–C. ON response components were characterized by the same PSTH parameters as described before. To characterize the OFF response component, response duration *(A2τ2)*, time to peak *(L2)* and response amplitude *(A2)* were extracted from PSTHs.

ON *(A1τ2)* and OFF *(A2τ2)* response durations were not significantly changed between NCBE KO (n = 15) and WT ON-OFF ganglion cells (n = 16) when the spot size was increased (Fig. 7D; for ANOVA values, please refer to the legend of Fig. 7). Interestingly, while response amplitudes did not differ between genotypes (*A1*, *A2*; Fig. 7F), the ON response component was slower in NCBE KO mice whereas the OFF response components was not (*L1*, *L2*; Fig. 7E).

Again, we used a spot size of 300 µm to stimulate ganglion cell receptive field centers and varied light intensity. Under these conditions, ON and OFF response durations of NCBE KO ON-OFF ganglion cells were significantly increased (*A1τ2*, *A2τ2;* Fig. 7G). As NCBE KO cells showed slower ON responses to light stimuli (*L1*; Fig. 7H, top), while OFF responses did not differ from NCBE WT (*L2*, Fig. 7H, bottom), we also analyzed the responses of NCBE KO ON-OFF ganglion cells to flicker stimuli (Fig. 7I) and used the maximal response amplitude (*A1*) as a measure. NCBE KO ON-OFF ganglion cells were able to follow temporal frequencies <5 Hz comparably to WT cells. However, ON-OFF ganglion cells from NCBE KO showed decreased responses to temporal frequencies between 5–10 Hz (Fig. 7I) and did not respond to stimuli above 10 Hz while WT cells followed flicker stimuli up to 15 Hz (Fig. 7I). These results are consistent with the ERG recordings, which showed similar frequency differences between genotypes (Fig. 5H).

In summary, PSTH analyses of ganglion cell light responses revealed that NCBE KO ON-OFF ganglion cells were slower than WT cells. In contrast, NCBE KO ON-transient ganglion cells were faster and showed more sustained responses than NCBE WT ganglion cells.

### Both Visual Acuity and Contrast Sensitivity were Impaired in NCBE KO Mice

**Figure 8:**
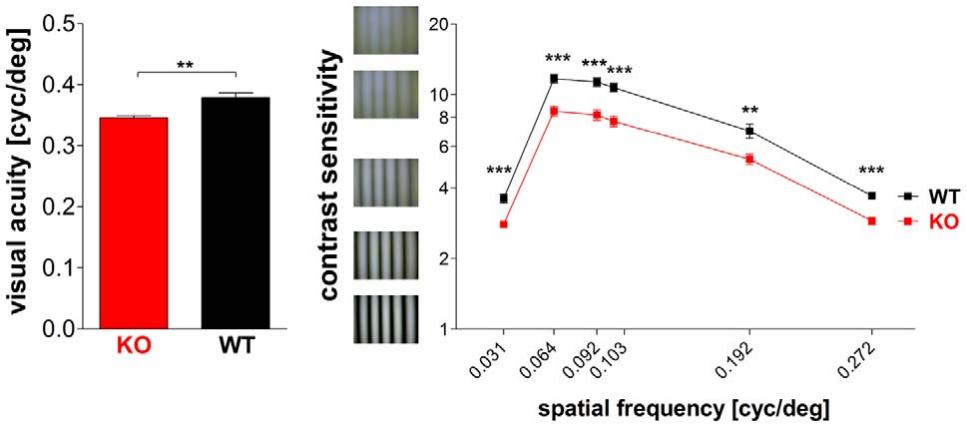
Impaired visual performance of NCBE-deficient mice. ***A,*** Visual acuity was significantly reduced in NCBE KO mice. The highest spatial frequencies eliciting an optomotor response were 0.346±0.008 cycles/degree (n = 7) in NCBE KO and 0.379±0.017 cycles/degree (n = 5) in NCBE WT mice. ***B,*** Michelson contrasts were plotted against the spatial frequencies in a log-log graph. Contrast sensitivity was significantly reduced in NCBE KO mice at all tested spatial frequencies. Statistical tests were performed with Student’s *t*-test (*p-value ≤0.05; **p-value ≤0.01; ***p-value ≤0.001) and values are presented as mean ± SEM.

To test whether the altered ganglion cell responses impair general visual function in NCBE KO mice, we measured both visual acuity and contrast sensitivity using a virtual-reality optomotor system [30], [31]. Visual acuity was quantified by increasing the spatial frequency of the moving full contrast gratings until an optokinetic response could no longer be determined. Our experiments revealed a small but significant difference (p = 0.0012, *t*-test) between the visual acuity of NCBE KO and WT mice (Fig. 8A): the highest spatial frequencies eliciting an optomotor response were 0.346±0.008 cycles/degree in NCBE KO (n = 7) and 0.379±0.017 cycles/degree in NCBE WT mice (n = 5), respectively.

Figure 8B shows contrast sensitivity values of NCBE KO and WT animals, measured in the optomotor setup and plotted as a function of spatial frequency. Contrast sensitivity of WT mice was clearly superior to the performance of the mutants. Statistical analyses confirmed that genotype had a significant impact on contrast sensitivity (p≤0.01, *t*-test): for instance, at a spatial frequency of 0.064 cycles/degree, NCBE KOs had a contrast sensitivity of 8.4±0.59 (corresponding to 11.9% contrast, n = 7) while WT littermates had a contrast sensitivity of 11.7±0.40 (or 8.6% contrast, n = 5). Contrast sensitivity in NCBE KO mice was reduced by a factor of 1.3 to 1.5 (at the different spatial frequencies) compared to WT littermates indicating that NCBE mice need approximately 1.5-fold higher contrasts in visual stimuli to trigger the optomotor reflex. Contrast sensitivity curves peaked at a spatial frequency of 0.064 cyc/deg for both NCBE KO and WT mice, as previously described for C57BL/6-mice [30].

## Discussion

Here, we show the subcellular distribution and the functional significance of the sodium-driven chloride bicarbonate exchanger NCBE (*Slc4a10*) in the adult mouse retina. Lack of NCBE leads to decreased acuity and contrast sensitivity, decreased ON bipolar cell activity, and temporal changes in light-evoked ON-transient and ON-OFF ganglion cell responses.

### NCBE Expression in KCC2-expressing Compartments of Retinal Neurons

Using different markers for bipolar cells [18], we show that NCBE is expressed in the dendrites of all OFF bipolar cell types with the exception of type 3B cells. Importantly, NCBE was never found on ON bipolar cell dendrites, whereas it is strongly expressed in ON and OFF bipolar cell somata and axon terminals. This differential NCBE expression remarkably resembles the expression pattern for the potassium chloride co-transporter KCC2. In line with previous studies, we found that KCC2 is expressed in both plexiform layers of the retina, predominantly in OFF bipolar cell dendrites and ON and OFF bipolar cell terminals [14], [32]. Double stainings confirmed almost a complete colocalization of NCBE and KCC2.

KCC2-positive bipolar cell compartments are known to show a hyperpolarizing response to activation of GABA_A/C_ receptors because their [Cl^−^]_i_ is low [14], [33] as KCC2 lowers [Cl^−^]_i_ in neurons [34]. Since NCBE most likely also acts as a chloride extruder [7], [8], [35], it may support KCC2 in maintaining a low [Cl^−^]_i_ in OFF bipolar cell dendrites and terminals and ON bipolar cell terminals and may thereby contribute to the hyperpolarizing action of GABA on these compartments [33], [36]. Consistent with this hypothesis, we did not find NCBE expression on compartments which are known to have a high [Cl^−^]_i_, i.e. ON bipolar cell dendrites and horizontal cells [33], [36]. These compartments have been shown to express the chloride intruder NKCC1 [14], [32], [37] and consequently GABA was shown to induce a depolarization in ON bipolar cell dendrites [38] and horizontal cells [36], [39]. However, ganglion cell somata and dendrites did not show NCBE expression although these compartments have been reported to express KCC2 [40]–[42]. Yet, this may be due to the very prominent NCBE immunoreactivity in bipolar cell terminals, which could mask NCBE expression in ganglion cell dendrites.

In addition, some calretinin- and calbindin-positive amacrine cells expressed NCBE. Among them are the starburst amacrine cells, which are involved in generating direction selectivity in certain ganglion cell types [23]. Gavrikov et al. [25] showed that KCC2 is expressed in the distal and NKCC2 in the proximal dendrites of rabbit starburst amacrine cells. Thus, similar to ON bipolar cells expressing NKCC1 in their dendrites and KCC2 in their terminals, starburst amacrine cells possess an inherent asymmetry in the distribution of chloride co-transporters along their dendrites, thereby establishing a [Cl^−^]_i_ gradient, which may underlie direction selectivity [25]. Though we could not test this directly, it is tempting to speculate that the expression of NCBE coincides with KCC2 expression also in distal dendrites of starburst amacrine cells.

As the involvement of chloride in the transport process of NCBE is a matter of debate [9], [35], it is possible that the transporter acts as an electroneutral sodium bicarbonate co-transporter (NBCn2) contributing only to pH_i_ regulation. If this is the case, its expression in the mouse retina suggests that it may complement the function of the electroneutral sodium bicarbonate co-transporter NBCn1 as NBCn1 is expressed in photoreceptors [4] which lack NCBE/NBCn2 (Fig. S1).

### Lack of NCBE Leads to Visual Impairments

Lack of NCBE caused a decrease in visual acuity and contrast sensitivity, impaired ON bipolar cell function and temporal changes in ganglion cell responses. However, as effects are diverse and different for different ganglion cell types, we can only speculate on the mechanisms that lead to visual impairments in mice lacking NCBE. In general, changes in retinal signal processing may result 1) from defects in pH_i_ regulation, potentially leading to reduced neuronal excitability [12] and 2) from changes in GABA-mediated inhibition as NCBE may not only act as a pH_i_ regulator [9] but may also extrude chloride (see above).

The observed changes in retinal signal processing may be caused by a defect in pH regulation, potentially leading to a reduced excitability of retinal neurons because neuronal activity leads to changes in pH: bicarbonate flux through ionotropic GABA and glycine receptors lowers pH_i_ and raises extracellular pH, thereby linking synaptic inhibition and pH regulation [6]. Since bicarbonate uptake is decreased in NCBE-deficient neurons, intracellular pH is decreased [12]. This may lead to a reduced excitability by a number of mechanisms [43], for instance by decreasing glutamate-induced responses [44]. Also, a decrease in pH_i_ may lead to reduced glutamate release: Sinning et al. [45] showed that the sodium-dependent chloride bicarbonate exchanger NDCBE (*Slc12a8*) is presynaptically expressed in glutamatergic synapses and lack of NDCBE leads to impaired release of glutamate in neurons. Thus, NCBE may play a similar role in bipolar cell somata and terminals. As a consequence, NCBE-deficient ON bipolar cells may have a reduced excitability or a reduced glutamatergic signal transmission, which may represent an explanation for the changes in ERGs. This is supported by previous studies showing that intracellular acidosis attenuates ERG b-wave amplitudes [46], [47]. The appearance of the ERG, particularly the flicker ERG (Fig. 5I, J), is very different if changes are induced by primary effects on photoreceptors [26], reinforcing the hypothesis of a synaptic or generic sensitivity loss in ON bipolar cells. As we found no NCBE expression in ON bipolar cell dendrites, a generic sensitivity loss appears more likely. Interestingly, the behavior of the ON-OFF ganglion cells (Fig. 7C, I) best correlated with the rod ERG b-wave changes observed (Fig. 5G, H) in comparison to the ON ganglion cells (Fig. 6C, I). This suggests that NCBE has differential effects on specific types of ON bipolar cells each of which selectively connects to transient ON or ON-OFF ganglion cells.

These effects are consistent with a role for NCBE as a pH_i_ regulator [9]. If NCBE also acts as a chloride extruder [7], [8], [35] changes in retinal signal processing may also result from changes in GABA-mediated inhibition. The activation of GABA_A/C_ receptors in KCC2-expressing neuronal compartments leads to chloride influx and bicarbonate efflux [43], [48], and down-regulation of KCC2 lowers the driving force for GABA-induced currents [34]. If NCBE contributes to chloride extrusion and bicarbonate intrusion in retinal neurons, lack of NCBE may similarly decrease the driving force for GABA, thereby altering GABA-mediated inhibition. This may have caused the following: a) in the outer and inner retina, GABAergic inhibition is thought to be responsible for the antagonistic center/surround organization of ganglion cell receptive fields [49]–[51]. Thus, loss of NCBE may cause alterations in receptive field organization. Indeed, we found changes in the spatial organization of receptive fields from ON-transient cells: NCBE KO showed slightly higher responses to large stimuli, suggesting that surround responses were weaker in these cells (Fig. 6F). Consistently, contrast sensitivity in NCBE KO mice was reduced, also pointing to a change in the spatial organization of ganglion cell receptive fields [52]. b) In addition, we found that the temporal structure of ganglion cell responses from NCBE KO mice was altered (Fig. 6, 7). The prolonged ON response duration in ganglion cells is consistent with impaired GABA-mediated inhibition because GABAergic signals from amacrine cells are thought to make bipolar cell and ganglion cell responses more transient [53], [54]. If NCBE was missing in ON bipolar cell axon terminals and potentially in ganglion cell dendrites, GABA-mediated inhibition would decrease and responses would become more sustained [53], [55]. This effect may also account for the failure of NCBE KO mice to follow stimulus frequencies above 10 Hz (Fig. 5).

However, GABA-mediated inhibition plays a minor role in OFF bipolar cells, in which glycine-mediated inhibition dominates [56]. As glycine-mediated inhibition may be similarly affected because glycine receptors are also permeable for both chloride and bicarbonate [36], [57], it seems surprising that we did not find any significant differences between the OFF responses of NCBE WT and KO ON-OFF ganglion cells (Fig. 7) or in the high frequency range of the dark-adapted flicker ERG. Yet, differences in ganglion cell responses may have been obscured by cell-to-cell variation. Moreover, physiological effects of NCBE loss are difficult to interpret given the broad retinal expression of NCBE and the complexity of retinal inhibitory circuits [56]. Also, we cannot exclude compensatory mechanisms leading to altered ion transporter kinetics or expression levels in NCBE-deficient mice. For example, increased KCC2 levels could have compensated the loss of NBCE, resulting in smaller changes in chloride homeostasis. Indeed, we found no alterations in KCC2 immunoreactivity in NCBE KO mice but subtle changes in expression levels may have been missed.

Most likely, the changes in retinal processing also led to the observed changes in visual behavior although we cannot exclude that reduced neuronal excitability in higher brain areas [12] contributes to the reduced contrast sensitivity in NCBE-deficient mice. However, the strong NCBE expression in starburst amacrine cells, which are indispensible for the optomotor response [58], together with the altered temporal structures of ON and ON-OFF ganglion cell light responses strongly suggest that NCBE deficiency impairs the processing of moving stimuli. Indeed, our behavioral measurements show that the spatial frequency sensitivity of the optokinetic response is significantly lower in NCBE KO mice compared to WT animals.

In conclusion, we show that the NCBE is differentially expressed in bipolar and amacrine cells of the mouse retina and that lack of NCBE leads to changes in retinal signal processing and impaired visual performance. These data suggest that NCBE contributes to pH_i_ regulation in retinal neurons and make a role for NCBE in the regulation of chloride-dependent inhibition very likely.

## Materials and Methods

All chemicals were obtained from Carl Roth GmbH (Karlsruhe, Germany) unless stated otherwise.

### Ethics Statement

All experiments were carried out in accordance with the institutional guidelines for animal welfare of the Universities of Oldenburg, Jena and Tübingen, following the standards described by the German animal protection law (*Tierschutzgesetz*). Killing of mice for tissue analysis and procedures of animal experimentation were approved by the animal welfare officer of the respective facility (University of Oldenburg, Jena and Tübingen) and the local authorities (University of Oldenburg: Niedersächsisches Landesamt für Verbraucherschutz und Lebensmittelsicherheit; Tübingen: Regierungspräsidium Tübingen).

### Animal Preparation

For experiments, we used NCBE (*Slc4a10*) knockout (KO) and NCBE wild-type (WT) mice [12]. Briefly, deletion of exon 12 of the *Slc4a10* gene, which encodes the first of the predicted 11–13 transmembrane spans of *Slc4a10*, leads to a frameshift and a premature termination codon in exon 13. Mice were kept under a 12 h light/dark cycle and were used at ages between 3–12 months for experiments. Mice were anesthetized with CO_2_ and killed by cervical dislocation. For extracellular recordings, mice were dark-adapted for three hours before sacrifice. Eyes were enucleated, cornea, lens and vitreous body removed, and the retina was prepared for further use. Dissections were performed either in 0.1 M phosphate buffer (PB; pH 7.4) for immunhistochemistry or in Ringer’s solution (110 mM NaCl, 2.5 mM KCl, 1 mM CaCl_2_, 1.6 mM MgCl_2_, 10 mM D-glucose, 22 mM NaHCO_3_, pH was maintained at 7.4 by aerating with 95% O_2_/5% CO_2_ mixed gas) for extracellular recordings.

### Immunohistochemistry and Fluorescent Image Acquisition

For vertical cryosections, mouse eyecups were fixed in 2% paraformaldehyde (PFA; Riedel de Haën, Seelze, Germany) in PB for 2×15 min at room temperature and washed with PB several times. Eyecups were then cryoprotected in 30% sucrose in PB overnight at 4°C and embedded in Cryoblock (Medite GmbH, Burgdorf, Germany) at −20°C. Vertical sections (∼25 µm) were cut on a cryostat (Bright, Huntingdon, UK) and collected on slides. Vertical sections were blocked with CTA (5% ChemiBLOCKER, Millipore, Billerica, MA, 0.5% Triton X-100 and 0.05% NaN_3_) for at least 60 min at room temperature. After the blocking procedure, vertical sections were incubated with primary antibodies in CTA overnight at 4°C. Incubation with secondary antibodies in CTA was carried out for 2 hours at room temperature. After washing several times with PB, sections were mounted in Vectashield (Vector Laboratories, Burlingame, CA). All incubations and washing procedures were performed in the dark. Primary antibodies used for immunohistochemistry are listed and described in Table 1. Secondary antibodies were conjugated to Alexa Fluor 488, 568, 647 (Invitrogen, Karlsruhe, Germany), Cy3, or Cy5 (Jackson ImmunoResearch Laboratory, West Grove, PA), respectively. Experiments, in which the primary antibodies were omitted, were performed to control for unspecific binding of the secondary antibody.

**Table 1:** Primary antibodies used in this study.

Antibody	Immunogen	Species, type, dilution	Source (Cat. No.)
Calbindin D-28K	Chicken calbindin D-28K, full-length amino acid sequence	Mouse, monoclonal, 1∶5,000	Swant, Marly, Switzerland (300)
Calretinin	Guinea-pig calretinin, full-length amino acidsequence	Goat, polyclonal, 1∶1,000	Millipore, Billerica, MA (AB1550)
ChAT	Human placental enzyme	Goat, polyclonal, 1∶250	Millipore, Billerica, MA (AB144P)
CSEN	Recombinant human calsenilin	Mouse, monoclonal, 1∶2,000	W. Wasco, Harvard Medical School, Charlestown, MA
Gα0	Bovine brain Gα0	Mouse, monoclonal,1∶250	Millipore, Billerica, MA (MAB3073; clone 2A)
HCN4γ	Mouse HCN4,amino acids 1,116–1,201	Guinea pig, polyclonal, 1∶1,000	F. Müller, Forschungszentrum Jülich, Germany
KCC2	Rat KCC2 (*Slc12a5),* amino acids 8–22	Rabbit, polyclonal, 1∶500	C. Hübner, University Hospital Jena, Germany
NCBE	Mouse NCBE (*Slc4a10*), amino acids 71–85	Rabbit, polyclonal, 1∶500	C. Hübner, University Hospital Jena, Germany
NCBE	Mouse NCBE *(Slc4a10),*amino acids 71–85	Guinea pig, polyclonal, 1∶500	C. Hübner, University Hospital Jena, Germany
NK3R	Rat neurokinin 3 receptor,amino acids 410–417	Rabbit polyclonal, 1∶500	A. Hirano, Geffen School of Medicine at UCLA, CA
PKARIIβ	Human protein kinase A regulatory subunit IIβ,amino acids 1–418	Mouse, monoclonal, 1∶2,000	BD Biosciences, San Jose, CA (610625)
PKCα	Purified bovine brain protein kinase C	Mouse, monoclonal, 1∶1,000	Sigma Aldrich, München, Germany (P 5704; Clone MC5)
ZNP-1	Synaptotagmin II, 1–5 day zebrafish embryo	Mouse, monoclonal,1∶500	Zebrafish International Resource Center (081002-25)

For hematoxylin/eosin (H/E) stainings eyecups were fixed in 4% PFA in PB overnight. Then the tissue was dehydrated, paraffin embedded and cut into 7 µm sections that were H/E stained following standard procedures as described previously [59].

Confocal micrographs of fluorescent specimen were taken with a Leica TCS SL confocal microscope (Leica, Wetzlar, Germany). Scanning was performed with a 63×/1.32 Plan-Apochromat objective at a resolution of 1,024×1,024 pixels. Scans of different wavelengths were done sequentially to rule out spill-over between red, green, and blue channels. Images are presented either as single scan sections or projections of 10–15 sections (∼5–7 µm). Images were superimposed and adjusted in brightness and contrast using Photoshop CS4 (Adobe, San Jose, CA). Colocalization analyses were performed as described earlier [60]. Briefly, we used the public domain Java image processing program ImageJ (NIH) and performed fluorescence intensity correlations to assess colocalization of NCBE with other markers.

### Electroretinography

Electroretinography was performed as described previously [27]. Mice were measured at PW4 (NCBE WT and KO, n = 4) and PW12 (NCBE WT, n = 2; NCBE KO, n = 3). Prior to experiments, mice were dark-adapted overnight. Anesthesia was induced by subcutaneous injection of ketamine (66.7 mg/kg body weight) and xylazine (11.7 mg/kg body weight). Short needle electrodes served as reference (forehead) and ground (tail) electrodes, and gold-wire ring electrodes as active electrodes. The ERG equipment consisted of a Ganzfeld bowl, a direct current amplifier, and a PC-based control and recording unit (Multiliner Vision; VIASYS Healthcare GmbH, Höchberg, Germany). Band-pass filter cutoff frequencies were 0.3 and 300 Hz, respectively. We obtained single-ﬂash ERG responses both under dark-adapted (scotopic) and light-adapted (photopic) conditions. Light adaptation was accomplished with a background illumination of 30 cd/m^2^ starting 10 min before photopic recording session. Stimuli were presented with increasing intensities, reaching from −4.0 to 1.5 log cd*s/m^2^ under scotopic and from −2.0 to 1.5 log cd*s/m^2^ under photopic conditions, divided into 10 and 8 steps, respectively. We also obtained responses to trains of ﬂashes (ﬂicker) using two fixed intensities (−2.0 and 0.5 log cd*s/m^2^) and 12 flash frequencies (0.5, 1, 2, 3, 5, 7, 10, 12, 15, 18, 20, and 30 Hz). The amplitude of the b-wave, which represents the activity of ON bipolar cells, was measured from the trough of the respective a-wave to the following peak of the b-wave [27]. The b-wave latency was defined as the time from flash onset to the peak of the b-wave.

### *In vivo* Imaging

Confocal scanning laser ophthalmoscopy (cSLO) images were obtained from anesthetized mice immediately after ERG recordings using a Heidelberg Retina Angiograph (HRA I) as reported previously [61], [62]. Spectral domain optical coherence tomography (SD-OCT) imaging was done in the same session as SLO, i.e. animals remained anaesthetized. Mouse eyes were subjected to OCT using a Spectralis™ HRA + OCT device from Heidelberg Engineering [62], [63]. Optical depth resolution is ca. 7 µm with digital resolution reaching 3.5 µm [62]. Imaging was performed using the proprietary software package Eye Explorer (version 3.2.1.0, Heidelberg Engineering).

### Extracellular Ganglion Cell Recordings

For these measurements, mice older than PW12 were used. Dissections were performed in the dark under dim red light [64]. Small incisions were made into the rim of the dissected eyecup to facilitate inversion. The eyecup was then inverted onto a balsa wood dome, stabilized with tissue paper, and transferred to the recording chamber. Ringer’s solution, warmed to 32°C using a heated cannula (MultiChannel Systems, Reutlingen, Germany), flowed continuously over the preparation from a small needle positioned at the apex of the inverted eyecup. After dissection, retinas were left to recover and dark-adapt for 1 h. 20 min before recordings, full-field flashes were presented (duration 300 ms, 1 Hz, 6.9 cd*s/m^2^) to assure identical light conditions for all retinas. Glass electrodes (Hilgenberg, Malsfeld, Germany) had resistances of 2–3 MΩ and were filled with Ringer’s solution. During the search for a cell, the retina was stimulated with light flashes (300 ms duration, 0.3 Hz, 6.9 cd*s/m^2^) while the light stimulus was centered on the electrode tip.

### Light Stimulation

Light stimuli were generated by a 100 W tungsten halogen lamp and intensity was controlled by a set of neutral density filters (Zeiss, Göttingen, Germany). We used stimuli with an increasing spot size (75–1,700 µm) and constant light intensity (6.9 cd*s/m^2^) and increasing intensities (−4.0 to 2.0 log cd*s/m^2^) with a constant spot size (300 µm). We recorded blocks of 2 s for each stimulus and repeated each block 5 times with an interval of 6 s between stimuli. Stimulus duration was 300 ms with a delay of 100 ms. For flicker stimuli (1–15 Hz) we used a constant spot size (300 µm) at a constant intensity (6.9 cd*s/m^2^). Stimulus duration and intervals between were aligned evenly with respect to the increasing frequency. We recorded blocks of 10 s for each flicker stimulus and repeated each block 5 times.

### Data Acquisition

Light-evoked signals were amplified (DAM-50, World Precision Instruments, Sarasota, FL), bandpass-filtered (10–10,000 Hz) and digitized with a sampling rate of 20 kHz by PowerLab/4SP (ADInstruments, Spechbach, Germany). Signals were recorded with Chart 5.5 (ADInstruments), digitally bandpass-filtered (300–5,000 Hz) and exported for spike sorting (Offline sorter 2.8.8, Plexon, Dallas, TX). Spikes were detected by threshold crossing using 2.8 sigmas of the standard deviation from the mean peak histogram. Cluster contours of the principle components were derived from the expectation maximization algorithm, in which the distribution of waveforms from each unit is modeled as a multivariate student’s t-distribution. Timestamps were exported to Neuroexplorer 3.266 (Nex, Littleton, MA) and a PSTH (bin width 1 ms) was calculated from the recorded 5 blocks for each stimulus. The PSTH was smoothed with a 25 ms Gaussian function to minimize alterations of the firing rate and to enhance signal-to-noise ratio.

### PSTH Parameters and Classification of Ganglion Cells

From the PSTH we extracted several parameters to describe the light responses of NCBE KO and WT ganglion cells [65]. For ON responses: the peak response after stimulus onset, corrected by the average baseline spike firing rate for the first 100 ms before stimulus onset, was taken to be the cell’s response amplitude *(A1)*. Time to peak *(L1)* was defined as the time from stimulus onset to *A1*. The parameter *A1τ2* estimates the cell’s firing time (response duration) from *A1* to a defined criterion response *(A1/e)*. For OFF responses, we similarly calculated the parameters *A2, L2* and *A2τ2* in relation to stimulus offset.

To classify cells into physiological response types, we followed the response classification after [65]. Briefly, PSTH parameters *A1* and *A2* were transformed to a bias index: *BI = (A1−A2)/(A1+A2).* If *BI* tended to +1, cells were classified as ON ganglion cells; if *BI* tended to −1, cells were classified as OFF ganglion cells. A *BI* near 0 classified the cells as ON-OFF ganglion cells. To distinguish between ON-transient and ON-sustained ganglion cells, the response duration *(A1τ2)* was transformed to a histogram and two groups separated. ON ganglion cells with *A1τ2*<200 ms were classified as ON-transient ganglion cells and with *A1τ2*>200 ms as ON-sustained ganglion cells.

Values are presented as mean ± standard error of the mean (SEM). Individual parameters of WT and KO ganglion cells were compared with a repeated measure (RM) two-way ANOVA.

### Behavioral Determination of Visual Acuity and Contrast Sensitivity

Visual acuity and contrast sensitivity were determined using the virtual-reality optomotor system developed by Prusky et al. [30]. Brieﬂy, freely moving animals were exposed to moving vertical sine wave gratings of various spatial frequencies and contrasts and will reﬂexively track the gratings by head movements as long as they can see the gratings. Spatial frequency at full contrast and contrast at six different spatial frequencies were varied by the experimenter until the threshold of tracking was determined [31]. The threshold for grating acuity and contrast thresholds were measured at the following six spatial frequencies: 0.031, 0.064, 0.092, 0.103, 0.192, 0.272 cycles/degree (cyc/deg). Contrast sensitivity was calculated at each spatial frequency as a Michelson contrast from the screen luminance *(max − min)/(max + min)* and the reciprocal of the threshold (black mean, 0.22 cd/m^2^; white mean, 152.13 cd/m^2^). Statistical tests were performed with Student’s *t*-test and values are presented as mean ± SEM.

## Supporting Information

Figure S1 NCBE is not expressed in photoreceptors. ***A***, Projection (2 µm) of a NCBE WT retinal section stained for NCBE (green) and PSD95 (magenta). ***B–D,*** PSD95-labeled photoreceptor bases (**C**) showed no colocalization (**D**) with NCBE (**B**) in single scans (0.5 µm) of NCBE WT retinal sections. Scale bars = 10 µm.
(TIF)

Figure S2 Electroretinography in NCBE-deficient mice (age 4 weeks). ***A, D,*** Representative single flash ERG recordings from NCBE WT (black) and KO (red) mice for increasing intensities under dark-adapted (***A***, scotopic) and light-adapted (***D***, photopic) conditions. ***B, C, E, F,*** Box-and-whisker plots of single flash ERG b-wave amplitudes (***B, E***) and latencies (***C, F***), plotted against flash intensity. Scotopic b-wave but not a-wave amplitudes in NCBE KO mice were reduced (***A,*** ** ***B***), and b-wave latencies (***C***) were increased compared to NCBE WT mice. *Inset* in ***C***: Overlay of scotopic single flash ERG response traces of NCBE WT (black) and NCBE KO (red) mice at −2.0 log cd*s/m^2^ intensity (arrow head). Scale bar: horizontal 50 ms, vertical 200 µV. Under photopic conditions, b-wave amplitudes (***D, E***) and b-wave latencies (***F***) of NCBE KO mice were similarly affected. ***G,*** ** ***I,*** Representative ERG recordings of a flicker frequency series (flash intensity ***G***: −2 log cd*s/m^2^; ***I***: 0.5 log cd*s/m^2^) under scotopic conditions. Flicker amplitudes (***H, J***) of NCBE KO mice decreased at much lower flash frequencies than that of WT controls. In all quantitative plots (***B, C, E, F, H, J***), boxes indicate the 25% and 75% quantile range, whiskers indicate the 5% and 95% quantiles, and solid lines connect the medians of the data. NCBE WT (n = 4), NCBE KO (n = 4).
(TIF)

Figure S3 *In vivo* retinal morphology in NCBE WT and KO mice. Fundus images of NCBE WT (***A***) and KO (***C***) mice obtained with 514 nm wavelength. Solid lines indicate the origin of the OCT scans in NCBE WT (***B***) and KO (***D***) mice. On the right side of the panels, blow ups of the retinal layering are also shown. No morphological differences between the two mouse genotypes were observed. Abbreviations: GC: ganglion cell layer; IPL: inner plexiform layer; INL: inner nuclear layer; OPL: outer plexiform layer; ONL: outer nuclear layer; IS/OS: inner segment outer segment boarder; RPE: retinal pigment epithelium.
(TIF)
