# Supplementary figures and images for: Lack of the Sodium-Driven Chloride Bicarbonate Exchanger NCBE Impairs Visual Function in the Mouse Retina
Source: PLoS One. 2012 Oct 9;7(10):e46155. doi: 10.1371/journal.pone.0046155 (PMC3467262; doi:10.1371/journal.pone.0046155)

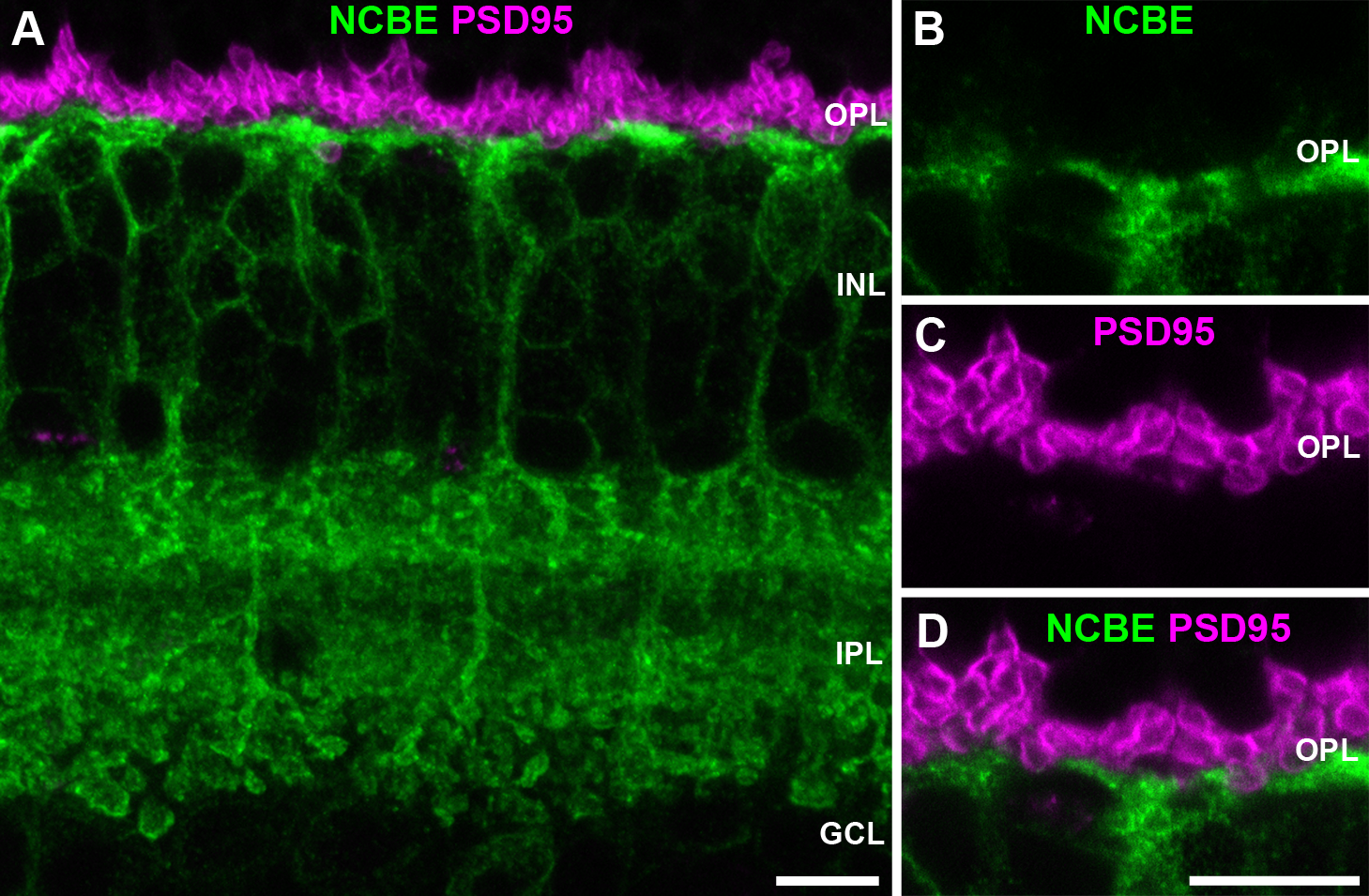

Supplement: Figure S1 — NCBE is not expressed in photoreceptors. A, Projection (2 µm) of a NCBE WT retinal section stained for NCBE (green) and PSD95 (magenta). B–D, PSD95-labeled photoreceptor bases (C) showed no colocalization (D) with NCBE (B) in single scans (0.5 µm) of NCBE WT retinal sections. Scale bars = 10 µm. (TIF) [file pone.0046155.s001.tif]

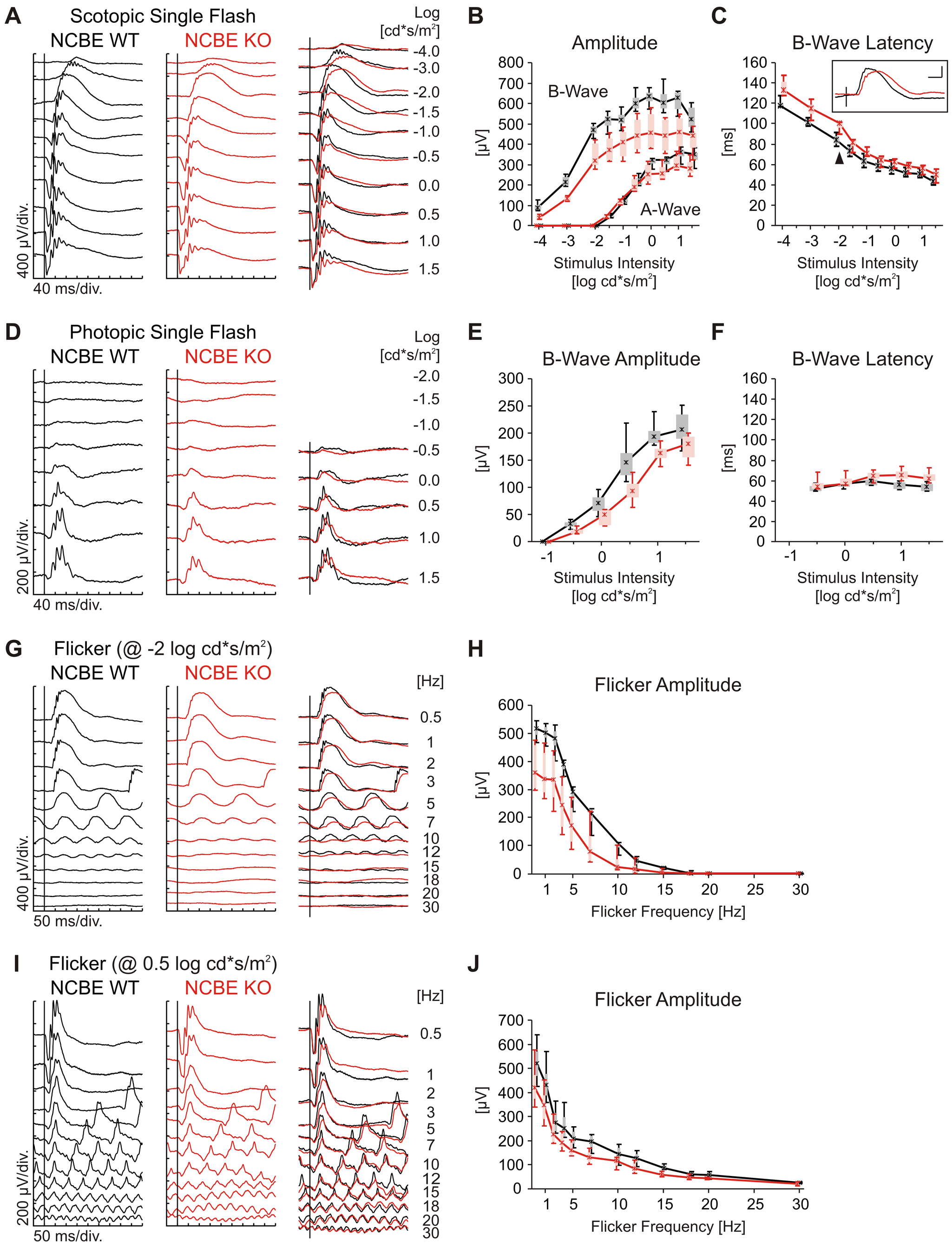

Supplement: Figure S2 — Electroretinography in NCBE-deficient mice (age 4 weeks). A, D, Representative single flash ERG recordings from NCBE WT (black) and KO (red) mice for increasing intensities under dark-adapted (A, scotopic) and light-adapted (D, photopic) conditions. B, C, E, F, Box-and-whisker plots of single flash ERG b-wave amplitudes (B, E) and latencies (C, F), plotted against flash intensity. Scotopic b-wave but not a-wave amplitudes in NCBE KO mice were reduced (A, B), and b-wave latencies (C) were increased compared to NCBE WT mice. Inset in C: Overlay of scotopic single flash ERG response traces of NCBE WT (black) and NCBE KO (red) mice at −2.0 log cd*s/m2 intensity (arrow head). Scale bar: horizontal 50 ms, vertical 200 µV. Under photopic conditions, b-wave amplitudes (D, E) and b-wave latencies (F) of NCBE KO mice were similarly affected. G, I, Representative ERG recordings of a flicker frequency series (flash intensity G: −2 log cd*s/m2; I: 0.5 log cd*s/m2) under scotopic conditions. Flicker amplitudes (H, J) of NCBE KO mice decreased at much lower flash frequencies than that of WT controls. In all quantitative plots (B, C, E, F, H, J), boxes indicate the 25% and 75% quantile range, whiskers indicate the 5% and 95% quantiles, and solid lines connect the medians of the data. NCBE WT (n = 4), NCBE KO (n = 4). (TIF) [file pone.0046155.s002.tif]

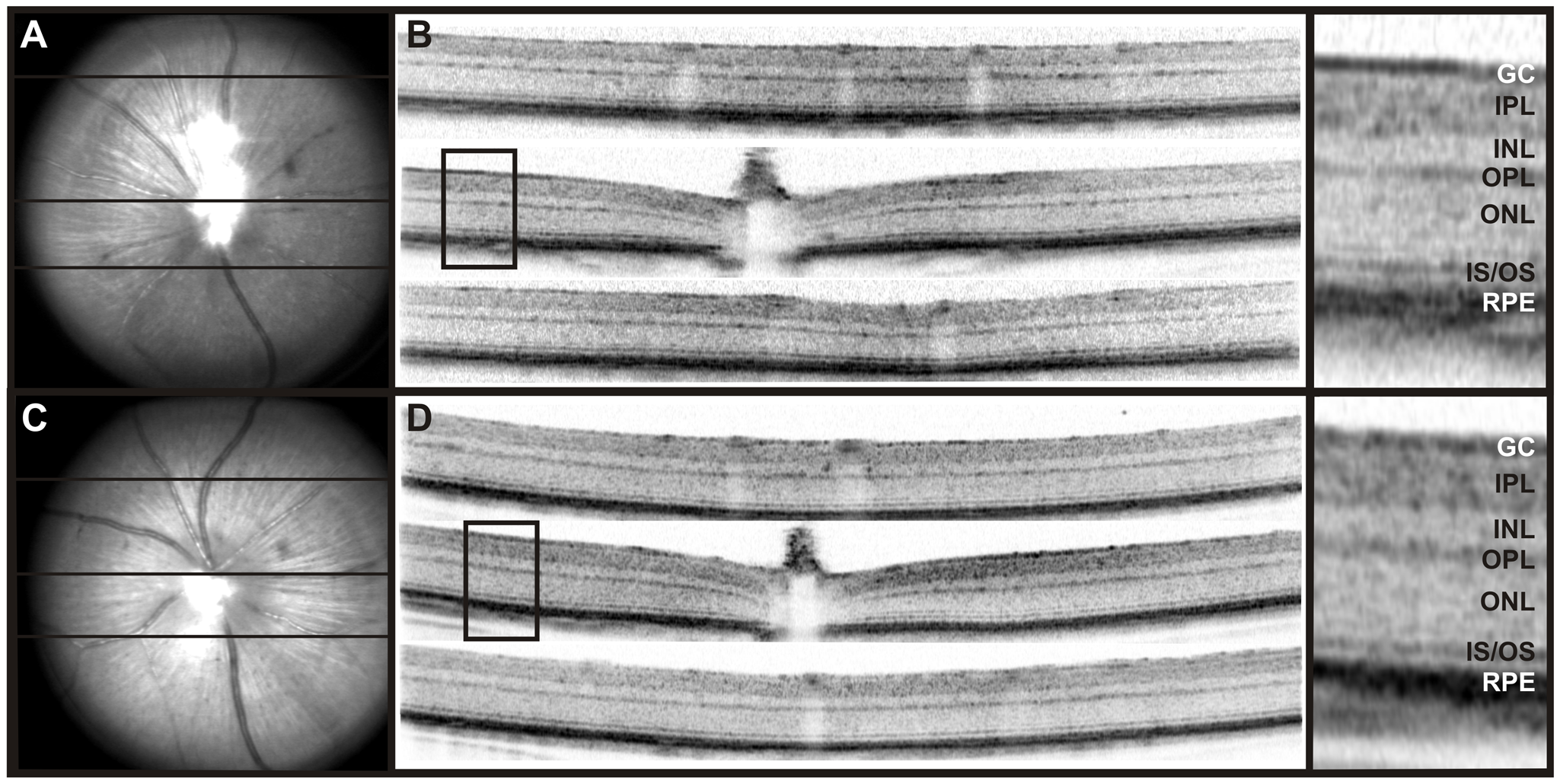

Supplement: Figure S3 — In vivo retinal morphology in NCBE WT and KO mice. Fundus images of NCBE WT (A) and KO (C) mice obtained with 514 nm wavelength. Solid lines indicate the origin of the OCT scans in NCBE WT (B) and KO (D) mice. On the right side of the panels, blow ups of the retinal layering are also shown. No morphological differences between the two mouse genotypes were observed. Abbreviations: GC: ganglion cell layer; IPL: inner plexiform layer; INL: inner nuclear layer; OPL: outer plexiform layer; ONL: outer nuclear layer; IS/OS: inner segment outer segment boarder; RPE: retinal pigment epithelium. (TIF) [file pone.0046155.s003.tif]
